# The association of leadership styles and nurses well-being: a cross-sectional study in healthcare settings

**DOI:** 10.11604/pamj.2020.36.328.19720

**Published:** 2020-08-24

**Authors:** Ibtissam Mohamad Sabbah, Tahanie Tarek Ibrahim, Rania Hani Khamis, Hajar Ahmad-Majed Bakhour, Sanaa Mohamad Sabbah, Nabil Sami Droubi, Hala Mohamad Sabbah

**Affiliations:** 1 Faculty of Public Health, Lebanese University, Saida, Lebanon,; 2 Institute of Social Science, Lebanese University, Saida, Lebanon,; 3 Doctoral School of Literature, Humanities and Social Sciences, Lebanese University, Beyrouth, Lebanon,; 4 Faculty of Economic Sciences and Business Administration, Lebanese University, Nabatieh, Lebanon

**Keywords:** Transformational leadership, SF-12v2 health survey, multifactor leadership questionnaire 5X short form, well-being

## Abstract

**Introduction:**

the nurses´ perception of their supervisors´ leadership styles has a substantial impact on their well-being. Effective leadership in health care is crucial in improving and enhancing the effectiveness of health care systems. This study aims to assess the leadership styles of nurse leaders as perceived by employees, and to explore the relationship between perceived leadership styles and the quality of life of nurses in Lebanese hospital settings.

**Methods:**

it was a cross-sectional study conducted in 2017 and involved a sample of 250 nurses chosen randomly in eight hospitals. The survey included questions on socio-demographic and health-related characteristics, Multifactor Leadership Questionnaire 5X Short Form, and the Short Form Health Survey-12 V2 (SF-12v2).

**Results:**

the managers used enough transformational leadership style, whereas they used fairly often transactional leadership. The Laissez-faire style was adopted from time to time by the managers. Male nurses perceive their managers as transformational significantly more than female nurses (2.94 vs. 2.73; p = 0.05). Transformational leadership style was statistically related to all scales scores of the SF-12v2 (p < 0.001) except the Social Functioning domain (p = 0.42). The transactional leadership style was associated with the Vitality scale scores (p < 0.001). The physical (p < 0.05) and Emotional Role (p < 0.001) and the mental health summary measure (p < 0.05) were lower in persons who perceived the leadership style of their manager as Laissez-faire.

**Conclusion:**

this study highlights the existence of a positive effect of leadership styles in the wellbeing of nurses, and confirms that nursing management has been identified as a challenge in the Lebanese hospitals.

## Introduction

The nurses´ perception of their supervisors´ leadership styles has a substantial impact on their working lives [1], health [2, 3] and well-being [4]. Employment is an essential element of an adult´s life, providing not only income but also a sense of engagement, role identification, physical and mental stimulation [5], and staff wellbeing in the modern workplace. The current challenges facing healthcare systems, in relation to the shortage of health professionals, require managers and leaders to learn different leadership styles and staff empowerment strategies [6]. Effective leadership in health care is crucial in improving and enhancing the effectiveness and efficiencies of health care systems [7]. Leadership has been described as the behavior of an individual when directing the activities of a group toward a shared goal [1]. Avolio and Bass [4] characterize the nurse leaders as transformational, transactional, and Laissez-faire. The transformational leaders encourage subordinates to view problems from new perspectives, provide support and encouragement, communicate a vision, and stimulate emotion and identification. Transactional leaders motivate subordinates through the use of contingent rewards, corrective actions and rule enforcement towards attaining common goals [4]. The non-leadership subscale was Laissez-faire.

Nurses are the main professional component of the front-line staff in most health systems. Nursing managers play a leading role in hospital management. Major studies demonstrate a positive relationship between managers´ leadership styles and staff retention [8-10], staff development [11], job satisfaction [7, 12-14], organizational [15-17] and nurses´ commitment [6], client satisfaction with nursing care [18] and lower levels of job stress and burnout [2]. The decentralized style of management, flexible employment opportunities, and access to continuing professional development can improve both the retention of nursing staff and patient care [8]. However, replacing nursing positions, such as the nurse manager, which requires strong leadership and at least minimal management experience and training, with less-qualified health care personnel has led to a devalued nursing role within organizations [6]. Integration of research evidence into clinical nursing practice helps nurses to provide high-quality patient care [19, 20], to meet development goals, and to deliver safe and effective care [8].

Nurses require leadership which provides direction for a new generation of nurses in their daily routine. Some will naturally adopt an effective leadership style, while others may find the concept of leadership or seeing themselves as leaders difficult to understand [11]. It is of paramount importance to explore and fully understand the potential determinants of psychological well-being in nurses [21-23]. Quality of life (QOL) concept has been recognized as a relevant measure of well-being in various healthy populations, including workers [24]. During the last decade, research on transformation and transactional leaderships significantly increased in Arabic countries [1, 6, 10, 13, 17, 18] and worldwide [7, 12, 14, 25-27]. Yet few studies have examined its potential positive effects on nurses´ well-being [22, 28]. Few researchers have studied the leadership characteristics of nurses managers in Lebanon [16, 29], and examined the quality of nurses´ work environment [30] and no attempt has been done to determine the relationship between management styles and nurses´ wellbeing in a hospital setting. The purpose of this cross-sectional, study is: (1) to assess the leadership styles of nurse leaders as perceived by employees; (2) to examine the degree to which the leadership characteristics correlate with specific outcomes of leadership behavior; and (3) to explore the relationship between perceived leadership styles and the quality of life of nurses at work.

## Methods

A cross-sectional study was conducted in the governorate of North and the South of Lebanon. The data collection was conducted using self-administered questionnaires between February and March 2017. Eight hospitals were selected to represent all Lebanese hospitals using a simple random sampling method. All consenting nurses working in hospitals in Tripoli and Saida were eligible to participate in the study. Inclusion criteria includes: 1) working as registered nurses, 2) has at least 3 months of experience at the current institution. A total of 260 were selected. Nurses were informed about the purpose of the study by a team of 2 trained interviewers and were invited to participate. All nurses gave their written consent to participate in the study. Also, participants remained anonymous and individual results were kept confidential. A pilot study including 10 individuals was performed previously in order to pre-test the feasibility of the questionnaire.

**Sample size:** the sample size was calculated according to Bartlett, Kotrlik, and Higgins table [31] using Cochran´s formulas, selecting an alpha level of 0.05, a margin of error of 0.05, and a population size approximately 8000 subjects (the total number of nurses working in hospital sector was 8853 [32]). The sample size needed equal 262.

**Data collection:** the study questionnaire had three sections.

**Characteristics of the respondents:** the first part of the questionnaire consisted of sociodemographic status, including age, gender, marital status, educational level, and job position. Data were also collected regarding the position of the direct leader, the type of hospital, the nursing department, work experience in nursing, nurse´s shifts, the intention to leave the work during the next six months, and declared morbidity.

**Multifactor Leadership Questionnaire 5X Short Form (MLQ 5X Short Form):** the second part consisted of the Arabic approved version of Multifactor Leadership Questionnaire 5X Short Form (MLQ 5X Short Form) to measure staff nurses´ opinions of their nurse managers. The questionnaire contains 45 items that measure nine characteristics of transformational, transactional, or passive/avoidant leadership styles and three outcomes of leadership behaviors, which are extra effort (EF), effectiveness (EFF), and satisfaction (SAT). Transformational leadership (TFL) includes the characteristics of idealized influence (attributes) (IIA), idealized influence (behaviors) (IIB), inspirational motivation (IM), intellectual stimulation (IS), and individual consideration (IC). Transactional leadership (TAL) includes characteristics of contingent reward (CR) and management-by-exception (active) (MBEA). Passive/avoidant (LFL) characteristics include management-by-exception (passive) (MBEP) and Laissez-faire leadership (LF) style. All of the leadership style scales have four items, Extra Effort has three items, Effectiveness has four items, and Satisfaction has two items. Each item is rated on a scale of 4 point Likert scale ranging from: 0 (not at all) to 4 (frequently, if not always) [4]. The MLQ 5X Short Form scale scores are average scores for the items on the scale. Blank answers should not be included in the calculation: if an item is left blank, divide the total for that scale by the number of items answered. The average of the five Transformational scale scores determines an overall Transformational score. A similar calculation with the TAL and LFL scale scores was performed. The perceived leadership styles, factors, and outcomes were interpreted as following: the mean range of 4.00-3.21 = frequently, if not always, from 3.20-2.41 = fairly often, 2.40-1.61 = sometimes, 1.60-0.81 = once in a while, and 0.80-0.00 = not at all. Since its development, the MLQ 5X Short Form has received extensive evidence of its reliability and validity, and is commonly used in leadership research [4]. The scale has good reliability with coefficients ranged from 0.73 to 0.94 [4]. The external validity of the MLQ 5X Short Form has also been established in previous studies conducted in health care organizations. In this study Cronbach alpha was 0.70 for all scales, except for active management by exception, which was 0.64 which demonstrates very good reliability (an alpha of greater than 0.50) [24].

**SF-12v2 Health Survey (SF-12v2):** the third part measuring wellbeing was the short form of the widely used 36-Item Short Form Health Survey (SF-36) called SF-12v2 Health Survey (Quality Metric Inc., Lincoln, RI, USA). Permission was taken from the Quality Metric Incorporated to use the Arabic version. It is a generic instrument, brief and reliable measure of overall quality of life (QOL) status. This questionnaire contains 12 items clustered to yield eight domains of QOL: physical functioning (PF), Physical Role (RP), Bodily Pain (BP), General Health (GH), Vitality (VT), Social Functioning (SF), Emotional Role (RE) and Mental Health (MH). The SF-12v2 provides a physical component summary (PCS-12) score as well as a mental component summary (MCS-12) score, which are derived from eight different subscales. The SF-12 was designed to give a population mean MCS and PCS of 50 with a standard deviation of 10 in a disease-free US population. We entered the responses of SF-12v2 for each patient into the SF website (SF Scoring Software V5 Setup). The Software computes the scores of all eight domains and the PCS-12 and MCS-12. Each response category has a score value; scores are summed across contributing questions and expressed as a score ranged from 0 to 100 for each dimension, as well as for the PCS-12 and MCS-12. A higher score indicates a better state of well-being.

**Statistical analysis**: descriptive statistical analysis such as frequency count, percentage, mean, median, and standard deviation (SD) was used to describe the research sample and responses to each scale. The differences between groups were tested with one way analysis of variance (ANOVA). Correlation between leadership styles, outcomes, and SF-12v2 scales score was analyzed using Spearman´s correlation. The scoring of the SF-12v2 was conducted using the SF Scoring Software V5 Setup. The SPSS 22.0 package (IBM SPSS Statistics, USA) was used for the analysis of the data. All tests of significance were two-tailed, p ≤ 0.05 was considered to be the critical level of significance.

## Results

Of the 260 nurses who received an invitation to participate, 250 (96.1%) completed a survey; 78.8% working in private hospitals.

**Demographics, socioeconomics, and health-related characteristics:** Table 1 showed that most of the participants (74.4%) were female, with a mean age of 29.3 years (SD = 12.0). Forty-four point eight percent were married. The mean years of professional experience in the current setting was 4.96 (SD = 4.75; minimum = 1, maximum = 27) years, 84.8% worked nurses for less than 10 years. The nurses worked in various wards. Sixteen point six percent (16.6%) decide, and 42.7% hesitant to leave work in the next 12 months. Twenty point four percent (20.4%) reported a disease or health problems.

**Table 1:** detailed characteristics of the study sample, and leadership styles according to the characteristics of the respondents (n = 250)

Variables	Frequency (%)	Leadership Styles: Mean (SD)		
		TFL	TAL	LFL
**Age**				
20- 29 years	142 (56.8)	2.80 (.70)	2.88 (.70)	1.49 (.83)
30- 39 years	90 (36.0)	2.81 (.81)	2.84 (.75)	1.58 (.83)
40 years and above	18 (7.2)	2.61 (.76)	2.72 (.75)	1.42 (.89)
**Gender**				
Male	64 (25.6)	2.94 (.70)	3.00 (.71)	1.53 (.90)
Female	186 (74.4)	2.73 (.75)‡	2.74 (.75)‡	1.52 (.81)
**Education**				
Baccalaureate	35 (14.0)	2.80 (.68)	2.81 (.74)	1.55 (.83)
Bachelor degree	181 (72.4)	2.74 (.76)	2.83 (.74)	1.58 (.81)
Master and Doctorate	34 (13.6)	3.00 (.66)	3.04 (.63)	1.16 (.86)‡
**Job position**				
Nurse	45 (18.0)	2.65 (.71)	2.73 (.71)	1.64 (.81)
Registered nurse	155 (62.0)	2.74 (.78)	2.82 (.75)	1.5 (.84)
Head Nurse	39 (15.6)	3.04 (.61)	3.08 (.63)	1.24 (.74)
supervisor	6 (2.4)	3.02 (.60)	3.04 (.57)	1.48 (.91)
Nursing director	5 (2.0)	3.44 (.64)‡	3.08 (.80)	1.05 (.90)
**Position of leader**				
Registered nurse	34 (13.6)	2.53 (.74)	2.56 (.80)	1.62 (.78)
Head Nurse	147 (58.8)	2.74 (.79)	2.84 (.74)	1.59 (.83)
Supervisor	21 (8.4)	2.86 (.54)	2.79 (.50)	1.17 (.78)
Nursing director	41 (16.4)	3.05 (.55)	3.14 (.61)	1.32 (.77)
General manager	7 (2.8)	3.21 (.76)†	3.02 (.84)†	1.61 (1.34)
**Nursing unit**				
Medicine & surgery	64 (25.6)	2.91 (.58)	2.94 (.65)	1.44 (.92)
Critical care unit	61 (24.4)	2.60 (.78)	2.71 (.74)	1.61 (.82)
Operating Room	20 (8.0)	2.36 (.87)	2.58 (.82)	1.49 (.80)
Maternity	46 (18.4)	3.02 (.73)	3.11 (.62)	1.34 (.73)
Emergency	13 (5.2)	3.13 (.45)	2.96 (.56)	1.35 (.63)
Pediatrics	20 (8.0)	2.39 (.85)	2.46 (.70)	1.86 (.70)
Others	26 (10.4)	2.95 (.66)*	2.97 (.81)†	1.77 (.94)
**Intent to leave work**				
Yes	26 (10.4)	2.51 (.82)	2.63 (.72)	1.61 (.76)
No	165 (66.0)	2.93 (.69)	2.97 (.71)	1.46 (.88)
No other choice	59 (23.6)	2.51 (.74)*	2.63 (.70)†	1.64 (.70)
**Disease**				
Yes	51 (20.4)	2.30 (.84)	2.44 (.83)	1.50 (.69)
No	199 (79.6)	2.91 (.66)*	2.96 (.66)*	1.52 (.87)
**Total sample**	250 (100)	**2.79 (.74)**	**2.85 (.73)**	**1.52 (.83)**

**Notes and abbreviations:** SD: Standard Deviation; TFL: Transformational leadership; TAL: Transactional leadership; LFL: Passive/avoidant; *: p is significant at the <.001 level (2-tailed); †: p is significant at the .01 level (2-tailed); ‡. P is significant at the .05 level (2-tailed). Blanks in table indicate a non-significant P value in that test.

**Perceived leadership styles, factors, and outcomes:** no-one refused to answer the questions of the MLQ 5X Short Form. The amount of missing data was very low, at only 0.04% of all answered items which indicate that the questionnaire had good acceptability. The mean (SD) transformational leadership score among the nurses who completed the MLQ 5X Short Form was 2.79 (0.74), whereas the mean (SD) transactional leadership score was 2.85 (0.73). Moreover, the lowest score was received by the passive/avoidant domain that was adopted from time to time (1.52, SD = 0.83) by the managers. However, nurses still had low scores of perception for the frequency of using theses styles where most of the mean of the transformational leadership and transactional leadership scales scores ranging from 3.20 to 2.41 (fairly often). Concerning leadership outcomes, nurses had fair perception in regards to extra efforts, effectiveness and satisfaction (Table 2). Comparing leadership style with those of the US normative population, the differences are summarized in Table 2. Briefly, the scores of all dimensions of MLQ 5X Short Form were similar to the American norms except for the MBEA, MBEP and Laissez-faire style of management, scoring higher means in this study than the United States norms, whereas the Laissez-faire style wasn´t adopted by Americans managers.

**Table 2:** descriptive statistics for MLQ 5X short form in comparison with an US normative sample

Lebanon (n=250)							US normative sample [4]		
	Mean	Median	SD	Skewness	Q1-Q3	Interpretation	Mean	SD	Interpretation
**TFL**	2.79	2.88	.75	-.50	2.3-3.35	Fairly often	2.83	-	Fairly often
**IIA**	2.89	3.00	.93	-.78	2.25-3.75	Fairly often	2.93	.82	Fairly often
**IIB**	2.67	2.75	.75	-.20	2.25-3.0	Fairly often	2.73	.76	Fairly often
**IM**	2.98	3.25	.85	-.71	2.25-3.75	Fairly often	2.97	.79	Fairly often
**IS**	2.80	2.75	.79	-.34	2.25-3.5	Fairly often	2.76	.75	Fairly often
**IC**	2.60	2.75	.92	-.39	2.0-3.25	Fairly often	2.78	.88	Fairly often
**TAL**	2.85	2.87	.73	-.36	2.38-3.50	Fairly often	2.27	-	Sometimes
**CR**	2.80	3.00	.84	-.42	2.25-3.50	Fairly often	2.84	.78	Fairly often
**MBEA**	2.90	3.00	.77	-.43	2.50-3.50	Fairly often	1.67	.92	Sometimes
**LFL**	1.52	1.44	.83	.32	.88-2.12	Once in a while	.84	-	Once in a while
**MBEP**	1.59	1.50	.92	.38	.75-2.25	Once in a while	1.02	.79	Once in a while
**LF**	1.45	1.50	.92	.21	.75-2.0	Once in a while	.66	.72	Not at all
**Outcomes**									
**EF**	2.73	2.67	.90	-.39	2.0-3.33	Fairly often	2.78	.94	Fairly often
**EFF**	2.87	3.00	.89	-.60	2.25-3.75	Fairly often	3.09	.78	Fairly often
**SAT**	2.85	3.00	.94	-.69	2.0-3.50	Fairly often	3.09	.91	Fairly often

**Abbreviations:** MLQ 5X Short Form: Multifactor Leadership Questionnaire 5X Short Form; US: United States; SD: Standard Deviation; Q1-Q3: first-third quartile; TFL: Transformational leadership; IIA: idealized influence (attributes); IIB: idealized influence (behaviors); IM: inspirational motivation; IS: intellectual stimulation; IC: individual consideration; TAL: Transactional leadership; CR: contingent reward; MBEA: management-by-exception (active); LFL: Passive/avoidant; MBEP: management-by-exception (passive); LF: Laissez-faire leadership; EF: extra effort; EFF: effectiveness; SAT: satisfaction.

**Quality of life (QOL) of study participants: SF-12v2 scale scores:** in assessing the quality of life of participants, the mean scores for scales measuring health related disability (PF, RP, BP, and RE) were higher than scores for scales measuring wellbeing (GH, VT, SF, and MH). The highest total mean score was for PF (70.10, SD = 31.58) and lowest for SF (55.90, SD = 30.52). The mean (SD) PCS and MCS scores, were respectively 48.66 (SD = 7.37) and 43.87 (SD = 9.11). Results showed that respectively 32.8% and 55.4% of participants scored lower on PCS-12 and MCS-12 scales than adult US population norms (Table 3).

**Table 3:** mean, SD, skewness, % floor and ceiling, quartiles of the SF-12v2 scale scores (n = 250)

	PF	RP	BP	GH	VT	SF	RE	MH	PCS-12	MCS-12
**Mean**	70.10	63.56	64.20	68.68	56.80	55.90	67.09	57.42	48.66	43.87
**SD**	31.58	25.08	26.76	22.47	26.20	30.52	26.14	19.94	7.37	9.11
**Skewness**	-.70	-.13	-.28	-.52	-.27	-.07	-.28	-.16	-.09	.05
**Minimum**	.00	.00	.00	.00	.00	.00	.00	.00	30.77	19.79
**Maximum**	100	100	100	100	100	100	100	100	65.40	66.62
**% floor**	7.2	1.6	2.8	.4	5.6	8.0	2.0	.4	.4	.4
**% ceiling**	43.2	18.8	23.2	14.8	11.2	19.6	25.2	4.8	.4	.4
**Quartiles**										
**25**	50.00	50.00	50.00	60.00	50.00	25.00	50.00	43.07	43.07	37.43
**50**	75.00	62.50	75.00	60.00	50.00	50.00	62.50	48.56	48.56	44.41
**75**	100	87.50	75.00	85.00	75.00	75.00	75.00	54.69	54.69	49.91

**Abbreviations:** SD: Standard Deviation; SF-12v2: SF-12v2 Health Survey; PF: physical functioning; RP: Physical Role; BP: Bodily Pain; GH: General Health; VT: Vitality; SF: Social Functioning; RE: Emotional Role; MH: Mental Health; PCS-12: physical component summary; MCS-12: mental component summary.

**Perceived leadership styles related to characteristics of nurses:** the analysis (Table 1) showed that MLQ 5X Short Form scores varied modestly across demographic and professional factors, with higher transformational leadership style and transformational leadership style scores among male (p < 0.05) and nurses getting a high level of education as master degree and above (p < 0.05). The Laissez-faire leadership was less adopted by the manager of nursing units with nurses getting a high level of education as master degree and above (mean score, 1.16; p < 0.05). Across wards, the transformational scores were highest in emergency department, and lowest in operating room (p < 0.001). A significant relationship was shown between the leadership styles of MLQ 5X Short Form and the position of the nursing unit's leader. The transformational style was more frequently (often, if not always) adopted by the General Manager (P < 0.01), whereas the transactional leadership style, the highest score was seen to be with the nursing director who followed fairly often this style of leadership (p < 0.01). Concerning age, marital status, type of hospital (public vs. private), shift work and length of work in nursing, the analysis showed that there were no significant differences between nurses´ perceptions of leadership styles and these variables (data not shown in tables). The intention to leave the wok within the next 6 months of the nurses´ respondents had a significant relationship with the transformational (p < 0.001) and transactional (p = 0.02) leadership style adopted by their managers at work. Finally the employees without morbidities reported that their nurse leaders displayed transactional and transformational leadership behaviors (p < 0.01).

**Perceived leadership styles related to outcomes and the quality of life of nurses:** Table 4 showed that there was positive and highly significant correlation between outcome factors (extra efforts, effectiveness, and satisfaction) and both transformational and transactional leadership styles (p < 0.001). The passive/avoidant leadership correlated negatively and weekly with extra efforts, and moderately with effectiveness and satisfaction (p < 0.001). Among nurses, well-being was associated with MLQ 5X Short Form scales scores. The transformational leadership style was statistically significantly associated to six of eight scales scores of SF-12v2 (RP, BP, GH, VT, RE, MH; p < 0.001); and both summary scores (p < 0.001). On the other hand, transactional leadership style influenced all SF-12v2 scales except PF (p = 0.42). The persons who perceived the leadership style of their manager as Laissez-faire had lower RP (p < 0.01), RE (p < O.001), BP (p < 0.05), and MCS-12 summary measure (p < 0.05) (Table 4).

**Table 4:** correlation between leadership styles, outcome factors, and the dimensions of SF-12v2: Spearman Rho correlations coefficients

Scales	Leadership>		
	TFL	TAL	LFL
**EF**	.79*	.69*	-.25*
**EFF**	.85*	.76*	-.33*
**SAT**	.82*	.77*	-.32*
**PF**	.07	.07	-.05
**RP**	.20*	.21*	-.19†
**BP**	.22*	.23†	-.13‡
**GH**	.18*	.16†	-.07
**VT**	.24*	.30*	-.03
**SF**	.06	.13‡	-.02
**RE**	.17*	.19†	-.24*
**MH**	.20*	.22*	-.01
**PCS-12**	.15*	.17*	-.09
**MCS-12**	.20*	.24*	-.13‡

**Notes and abbreviations:** SF-12v2: SF-12v2 Health Survey; TFL: Transformational leadership; TAL: Transactional leadership; LFL: Passive/avoidant; EF: extra effort; EFF: effectiveness; SAT: satisfaction; PF: physical functioning; RP: Physical Role; BP: Bodily Pain; GH: General Health; VT: Vitality; SF: Social Functioning; RE: Emotional Role; MH: Mental Health; PCS-12: physical component summary; MCS-12: mental component summary; *: Correlation is significant at the <.001 level (2-tailed); †: Correlation is significant at the .01 level (2-tailed); ‡. Correlation is significant at the .05 level (2-tailed). Blanks in table indicate a non-significant P value in that test.

## Discussion

Our study contributes to establish linkages between leadership styles and the quality of life of the nurses in healthcare settings. The results reveal that the transformational style of leadership contributed to an increase the quality of life and the outcome variables (extra effort, perceived leader effectiveness, and satisfaction) among nurses directly more than the other two leadership styles. As previously noted in the literature, our findings support that the leadership styles are associated with a better quality of life of nurses in the workplace, increase the satisfaction at work [7, 12-14], reduce the employee turnover level [8-10], may motivate employees to accomplish change [33], and can increase employee performance. Facilities whose leaders adopt transformational leadership have the ability to motivate their staff compared with those whose leaders adopted transactional leadership [34]. In contrast, the Laissez-faire leadership which avoids making decisions, delays actions, and ignores leader responsibilities leads to job dissatisfaction [33] and poor mental health. According to the MLQ 5X Short Form results, nurses perceived their managers as transformational as well as transactional leaders simultaneously. The total scores for the transformational and transactional leadership subscales are less than what have been studied by Avolio and Bass [4] which consider the optimum rating for transformational leadership be greater or equal three. Our results are higher than those in Saudi Arabia [6]. This result is concordant with previous studies [6, 7, 18]. Like the study by Yahchouchi *et al*. [16], we found that the leaders adopt the transformational style in managing their care units, relying rather on stimulation by motivating nurses in their directions (IM = 2.98). Therefore, the more subordinates perceived their leaders to be transformational, the more likely they were motivated [34], and achieved a high level of commitment through empowerment strategies and meaningful participation in decision-making [6]. Nevertheless, when subordinates perceived their leaders to use passive management-by-exception, subordinates tended to lose their enthusiasm [34]. Indeed, the transactional leadership style is an essential precondition for transformational leadership style as it helps to strengthen the relationship between the subordinates and the leader [6].

There are gender differences in perception of leadership behaviors. Male nurses perceive their managers as transformational leadership style more than female nurses. As reported by Aboshaiqah *et al*. [18], males were also more likely than females to report that their supervisors pay attention on the fulfillment of contractual obligations that include setting objectives, monitoring, and controlling outcomes. Our results do not provide a relationship between leadership styles and work experience. Several studies had found that older nurse managers had better skills and knowledge in leadership style than younger nurse managers [27]. This is likely due to the fact that when nurse managers have worked longer in the organizations, they have become acquainted with the strategy and vision of the organization and may have participated in creating the vision [27]. The position of leader as Registered Nurse had the lowest transformational scores. As in Finland, nurse managers usually have nurse education, specialized nurse education, and/or academic education. Nurse managers need updating education to develop their own professional abilities. Moreover, they need knowledge of nursing science and practice so as to be able to manage the work unit as a whole, and should know how to argue decisions in order to get employees to commit to their work and changes [27]. Varghese *et al*. [35] reported that the absence of quantifiable evidence on the nurse leadership crisis and the treatment of nursing reforms as a “second class” issue were found to negatively influence perceptions of the validity of nurse leadership reform. The present investigation confirms that nurses have a poor mental health which can affect patient safety, quality of care, and the profitability of the organizations [23]. It also leads to the burnout that is particularly common and severe among working nurses in Lebanon [3]. Similarly, employment may affect both physical and psychological health as well as leisure, and social participation [5]. Finally, we have shown overreporting of health problems among nurses providing evidence that psychological pain could be at the origin of somatic and psychological disorders [36].

The subordinates tend to have a higher level of quality of life when they perceive leader support as a specific behavior of transformational leadership styles. In our study, transformational leadership style plays an important role in the mental health of their subordinates. Nevertheless, the MH scores decreased with increasing passive leadership scores. Tountas *et al*. [28] reported that the poor health status and QOL of the nurses may primarily reflect the difficulties they face in their everyday life, and most importantly reflects the difficulty of being a woman working a job with a low professional (and consequently social) status in a predominantly male, highly competitive environment. Meng *et al*. [37], also suggest that nurses often work in a passive, subordinate position associated with increased respect toward doctors. In addition, the lack of strong support from nurses themselves for these policy reforms to strengthen the nursing sector is attributed to social disempowerment, and lack of professional autonomy [35]. Under such circumstances, nurses are treated with apathy and may even be manhandled or disrespected [37]. Nurses work a highly stressful and demanding profession which is not very well rewarded [28, 32]. This may explain the low scores of the SF-12v2 of nurses. Managers should encourage the idea of life-work balance not work-life balance (i.e. life is first) [38], and should adopt a more multidisciplinary approach to be effective leaders [39]. As reported by Aboshaiqah *et al*. [18], the study supports the notion that leadership styles can be combined to produce extra effort from subordinates as well as leadership effectiveness and satisfaction There were some potential limitations of the current study. It was carried out in only two areas of Lebanon because lack of resources limits the results to certain regions. Also, this is a cross sectional study which cannot establish a temporal relationship between variables and outcomes. Another limitation of this study is the sample size. While it was adequate for this analysis, it is insufficient to allow for a more detailed analysis of differences in quality of life in workplace and leadership styles across different hospitals´ departments. Some of the SF-12v2 subdomains were skewed which is to be expected in a general population. However, the parametric techniques that were sometimes applied are quite robust against skewness and have been demonstrated to be adequate in analyzing skewed data if sample size is large enough [40]. Blanca *et al*. [41] provided empirical evidence for the robustness of ANOVA under a wide variety of conditions involving non-normal distributions. In addition, the proportion of female nurses is very high, and similar to the gender distribution of nursing reported in Lebanon that remains steady at 80% [32].

## Conclusion

In summary, in this cross-sectional study, the wellbeing of nurses was positively associated with transformational and transactional leadership style adopted by their managers, and confirms that nursing management has been identified as a challenge in the Lebanese hospitals. Although, maintaining and strengthening well-being of nurses at work is important, nurses´ perspectives must be denoted at the highest levels of hospital leadership and incorporated into hospital decision making. The disparity between government and private health care settings, and females and male leadership continues to be an issue of debate and warrants more in-depth investigation. By training and rewarding managers to adopt transformational leadership behaviours, nursing leaders in hospital settings can improve the quality of life and the satisfaction at work for all nurses, and encourage nurses to remain active in Lebanon.

### What is known about this topic

- Effective leadership in health care is crucial in improving and enhancing the effectiveness of health care systems;
- Nurses´ perception of their managers´ leadership styles has a substantial impact on their working lives;
- Nurses require leadership which provides direction for a new generation of nurses in their daily routine.

### What this study adds

- Provides knowledge about leadership styles adopted in healthcare setting;
- Contributes to the existing literature on the effect of leadership style on nursing well-being and outcomes;
- Inform decision makers that training and rewarding managers to adopt transformational leadership behaviours can improve the quality of life and the satisfaction at work for all nurses.
